# Experience with Treating Lentigo Maligna with Definitive Radiotherapy

**DOI:** 10.1155/2018/7439807

**Published:** 2018-07-15

**Authors:** Gerald B. Fogarty, Angela Hong, Alex Economides, Pascale Guitera

**Affiliations:** ^1^Melanoma Institute Australia, Poche Centre, Crows Nest, Australia; ^2^Genesis Cancer Care, Mater Hospital, Crows Nest, Australia; ^3^Genesis Cancer Care, St. Vincent's Hospital, Darlinghurst, Australia; ^4^Australia and New Zealand Melanoma Trials Group, Sydney, Australia

## Abstract

Lentigo maligna (LM) is a form of melanoma in situ that occurs on exposed, sun-damaged skin; LM can progress to invasive melanoma. Conventional surgical treatment is the preferred management option as it is usually a one-treatment episode and generates a histopathology report that records completion of excision. Some patients may not be surgical candidates due to comorbidities, patient preference, impact on function, and cosmesis or they have failed surgery with a positive margin. Other therapies, including radiotherapy (RT) and topical medicines, may then become appropriate. There is a currently accruing multi-institutional randomized trial of imiquimod versus definitive RT for this population (NCT02394132). This review is about the experience from the centre that has generated the trial and enrolled the most patients to date. The purpose of the review is to pass on experience to other centers who may want to join the trial, especially to supplement the experience of local radiation oncologists. The review covers decisions that need to be made in RT planning and treatment and how to manage side effects and other common scenarios including LM in immunosuppressed patients and in poorly vascularised tissue, after surgery, of the eyelid and of mucous membrane (mouth and nose) that are in the radiation field.

## 1. Introduction

Lentigo maligna (LM) is a form of melanoma in situ that occurs on exposed sun-damaged skin of elderly people [1]. Australia has the highest incidence of LM in the world. With an aging population, LM rates are likely to increase [2]. There is an estimated 5% lifetime risk of progression from LM to invasive melanoma [3]. There is a range of evidence that demonstrates that 5mm surgical margins on the visible lesion are inadequate for excising LM in up to 50% of cases [4]

Lentigo maligna can be treated by radiotherapy (RT) in a definitive manner; the authors completed a literature review of previously published data for LM treated with definitive primary RT [5] which noted that, after a median of reported follow-up of 3 years, there was an LM recurrence rate of 5%. RT is usually for patients who are unable to have a surgical solution because of comorbidities, patient preference, impact on function, and cosmesis or who have already failed a surgery with positive margin with further surgery not being possible.

## 2. Decisions That Radiation Oncologists Need to Make in the Radiotherapy of LM

In determining how to treat a patient with lentigo maligna with radiotherapy, radiation oncologists (ROs) have a number of decisions to make. The decisions include whether to treat with radiotherapy or not and what is the intent of therapy.

If radiotherapy is to be prescribed and given, certain questions need to be asked at the planning phase. These include what volumes are to be treated and what volumes are to be avoided; what modalities in that particular department are available to give the best conformality of dose to these volumes; what the total dose and fractionation pattern should be. Simulation is an important part of planning that the RO should be present for ideally.

When the plan is accepted and treatment begins, the RO has to make the decision about what should be the intensity of the on-treatment reviews and ongoing support of the patient and staff.

When the treatment is finished, the RO also needs to be involved in the decision about how that patient should be followed up.

### 2.1. To Treat with Radiotherapy or Not

The decision to treat with radiotherapy or not ideally should be determined through an open discussion in the Multidisciplinary Clinic (MDC). It needs to be decided whether this particular patient is beyond surgery. MDC organization covers a wide range. Some units have a special Lentigo Maligna Clinic with all the treating specialists and the patient present. The most important aspect of the MDC is clear, concise, and prompt communication between all the members of the team, allied health included, aimed at delivering the best quality care to the patient; this can be either face to face or via a virtual meeting.

## 3. The Radiotherapy Treatment Decision Will Be Impacted by a Number of Factors

### 3.1. Patient Factors

These include, for example, the patient's performance status, projected life expectancy, and ability to attend a fractionated course of treatment. The latter will depend on mobility, the proximity of their place of residence to the treatment centre, and transport available to them. This will also be affected by their ability to receive treatment; for example, patients suffering claustrophobia may not be able to handle a RT immobilisation mask. Those suffering from dementia can also find adopting the treatment position for the duration of the beam-on time challenging. Those living alone may have insufficient support for daily dressings if and when needed. They may also be at risk of having nutritional issues if and when they become tired towards the end of the treatment process. Rarely, radiation sensitivity syndromes can also preclude patients from having radiotherapy.

### 3.2. Treatment Factors

Treatment factors also impact whether radiotherapy can be given or not. This will depend on the patient's fitness for other therapies, for example, Aldara (imiquimod cream), and their ability to apply it. It will also be impacted by whether the patient is immunosuppressed or not; this may change the way radiotherapy is delivered especially in terms of total dose. It may also impact on whether they are an imiquimod candidate given that imiquimod does work by enhancing the immune system. They may not be a surgical candidate for reasons such as being on life-sustaining anticoagulation; they may also want to avoid the functional and cosmetic impact of the necessary tissue loss that always happens with surgery. Surgery may have already failed with a positive margin without further tissue being able to be taken.

Previous therapies also impact radiotherapy decisions for treatment. This is particularly so if previous radiotherapy has been given to this area. Previous surgery can render the area hypoxic and can also drive lentigo maligna tissue deeper into the patient's flesh, for example, if a flap has been put over a positive margin.

### 3.3. Tumour Factors

Tumour factors also impact on whether radiotherapy should be prescribed or not. Factors such as the size of the lesion and the proximity of the lesion to radiation sensitive structures; for example, a lesion extending onto the bulbar conjunctiva will also impact whether radiotherapy can be given and if radiotherapy is to be given, then it may impact how that radiotherapy is delivered.

## 4. The Intent of Therapy

The next decision is what the intent of therapy should be. The intent of therapy for lentigo maligna is radical intent; it is either given in a definitive manner as the sole treatment, or adjuvantly, following incomplete surgery. The aim of therapy is local control and avoidance of progression to invasive disease. The only data about the risk of transformation of lentigo maligna into invasive melanoma is from case studies. Some of these case studies have shown there is a lifetime risk of up to 50% of malignant transformation with the possibility of distant disease and death [3, 4, 6–10]. The usual practice is that patients with a healthy life expectancy are treated.

**Figure 1:**
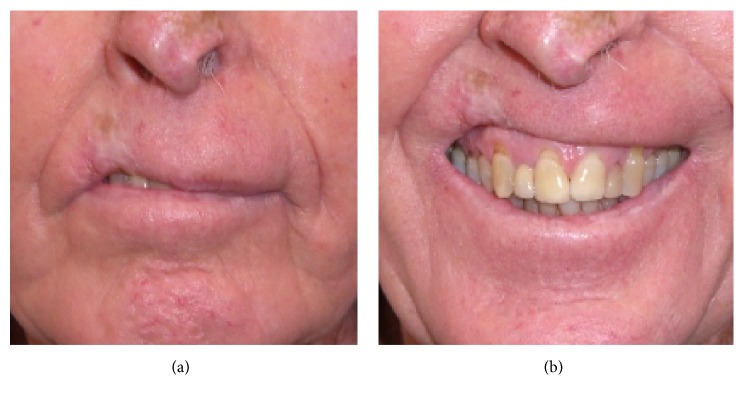
(a) Lip lesion treated successfully with definitive radiotherapy but with hypofractionation causing the late effects of hypopigmentation, telangiectasia, and cicatrization. (b) The irradiated tissue does not move as well as the other tissue, ruining this gentleman's smile.

Some patients can have significant comorbidities but will still live for some years. This means that in prescribing radiotherapy an appropriate fractionation pattern that will avoid radiation late effects has to be factored in. It is very important that the quality of life of these people after irradiation is better than if radiotherapy was not given. Radiotherapy given in large dose per fraction can cause death of normal cells, swamping their radiation repair mechanisms, and those normal cells are eventually replaced with fibrous tissue. This results in hypopigmentation, telangiectasia and cicatrization of the radiation field (see Figure 1), and hyperpigmentation. Fractionation does avoid the delayed effects of fibrosis

## 5. Planning

Once radiotherapy has been decided upon as treatment, the patient needs to proceed to planning. Planning involves a number of steps.

### 5.1. Volume Considerations

Radiotherapy treats a volume of tissue to a dose. Volume is more important than dose. If the wrong dose is given to the right volume, at least some treatment has been given to the appropriate volume but if the right dose is given to the wrong volume, then the patient ends up with side effects in normal tissue and a completely untreated cancer.

In lentigo maligna, the volume is defined by an area of skin and a depth to the bottom of the appendageal structures in the dermis which includes hair follicles and sweat glands. The area is usually larger than the pigment visible to the naked eye; underestimation of this fact has led to field edge recurrences in the past [5].

When treating with radiotherapy it is important to follow recommendations in volume definition from ICRU 50 [11]; the gross tumour volume (GTV) is the pigmentation that is visible to the naked eye. The clinical target volume (CTV) is the area that has been mapped with the use of imaging which can include reflectance confocal microscopy (RCM), dermoscopy, and targeted biopsy. The planning target volume (PTV) depends on the modality of treatment used and on the effectiveness of immobilisation protocols in that particular department.

In defining these volumes, particularly the CTV, there is a reliance on other specialties, particularly dermatologists. Therefore, there is a need for clear communication of treatment areas between the specialists and all involved in the follow-up of this patient. A convenient way to do this is by sharing the templates that are produced at simulation.

**Figure 2:**
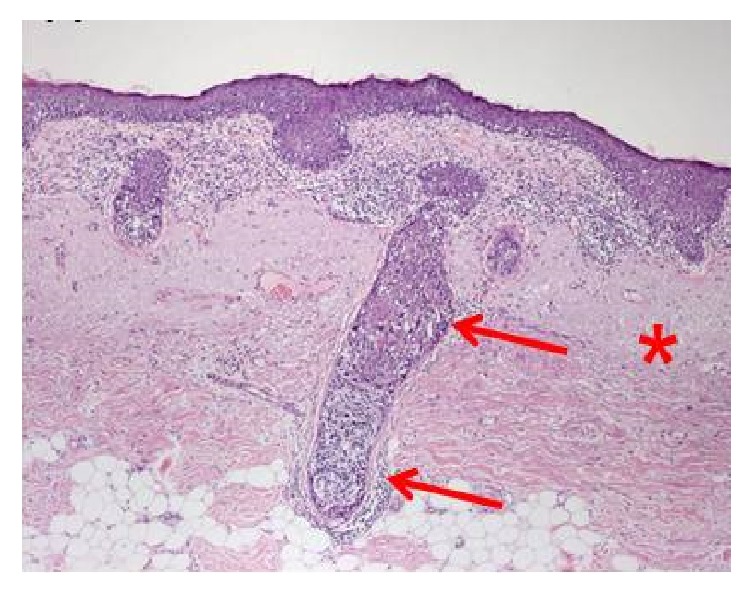
Skin target of lentigo maligna. The radiotherapy dose needs to treat 5 mm depth as lentigo maligna can invade down adnexal structures causing in-field failure.

The depth that needs to be treated has been shown to be a maximum of 4.5 mm [5]. (See Figure 2.) Therefore, it is important to plan a meaningful dose of radiotherapy to 5 mm from the skin surface or to where there is an oncological barrier, e.g., skull. This depth may need to be compromised on when the tissue to be treated is close to vital structures, for example, the lacrimal gland.

**Figure 3:**
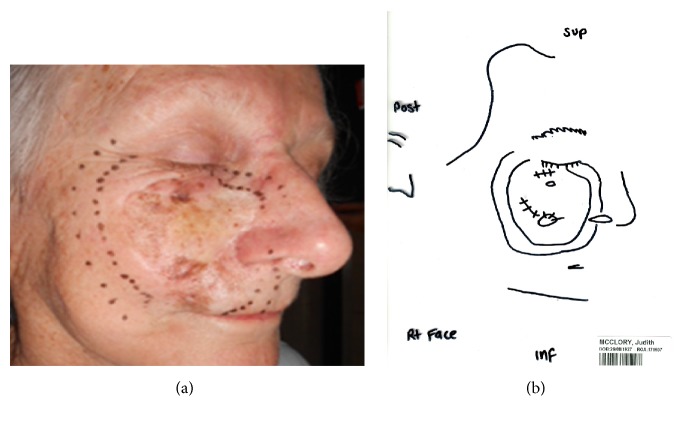
(a) This lady had a large area of lentigo maligna that has failed a surgical graft mapped out on her right medial cheek. The radiation oncologist has duplicated the RCM area onto that face. At least a 1 cm margin is then put around that area to act as field edge. (b) The radiation therapists have then produced a template on a piece of plastic which will be used to transfer marks onto the mask and will be used every day for treatment verification on the machine. This piece of plastic can be stored in an electronic medical records system and be emailed to a dermatologist or any other caring doctor in the future if the radiotherapy area is ever needed to be delineated or known, for example, if there is recurrence of lentigo maligna and the team wants to know whether the lentigo maligna recurrence is in-field or not or a field edge recurrence.

Planning includes the process where these volumes are delineated. This is illustrated with the example (see Figure 3) of a large right cheek that has failed surgery. This lesion is mapped with RCM by the dermatologist. The patient was consented and a planning appointment was made. At planning the radiation oncologist reproduced the RCM area on the patient's face. A template was made of that area. A mask was made for the patient; the template was used to put the area defined by the template on to the mask; the lines on the mask were then marked out with wire so that during the planning scan the marks could be captured by the scanning.

### 5.2. Modalities

**Figure 4:**
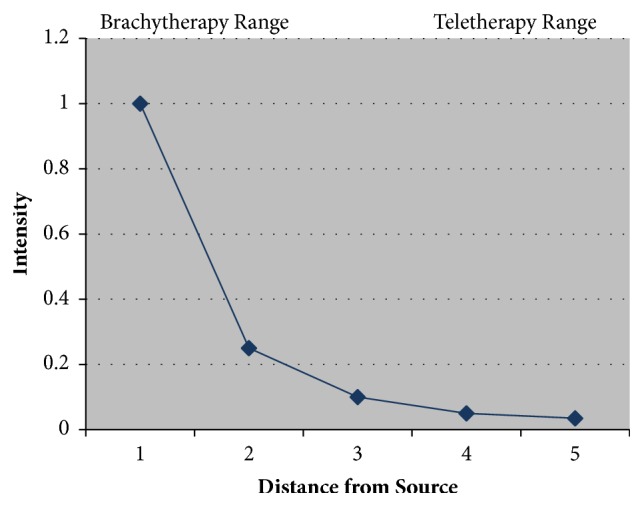
Radiotherapy modalities include external beam radiotherapy (EBRT) or brachytherapy (BT). These two classes of modalities are related by the inverse square law. Inverse square law states that as one moves away from the radioactive source, the amount of radiotherapy that can be measured is inversely proportional to the square of the distance.

The volumes will determine what modalities of radiotherapy can be used. Not every department has every modality and so some compromises may need to be made. Radiotherapy modalities include external beam radiotherapy (EBRT) or brachytherapy (BT). These two classes of modalities are related by the inverse square law (see Figure 4). Inverse square law states that as one moves away from the radioactive source, the amount of radiotherapy that can be measured is inversely proportional to the square of the distance.

**Figure 5:**
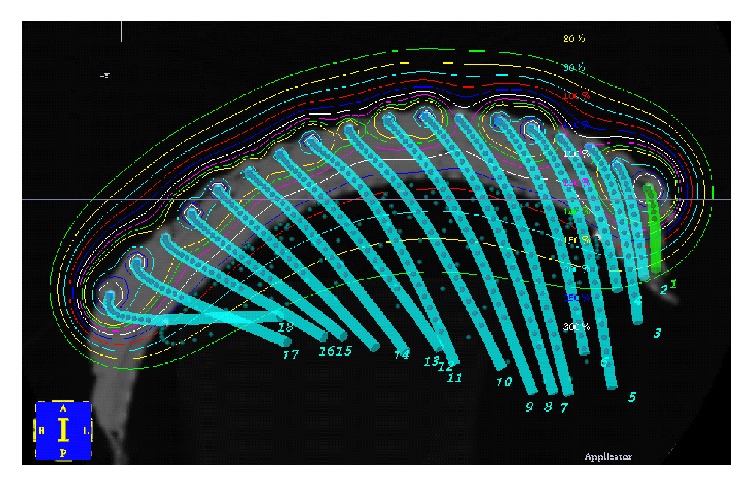
A brachytherapy mould where the brachytherapy containers or tubes or wires are kept in a pattern by the mould, usually the wires are separated by equal spacing. There is usually some water-equivalent material called bolus or build-up (BU) between the brachytherapy tubes and the skin; this enables the dose cloud, by the time it gets to the skin, to have a homogeneous wave front. See the thin red line.

Brachytherapy involves the application of a brachytherapy mould containing radioactive sources on to the skin. This is done via a brachytherapy mould where the brachytherapy containers or tubes or wires are kept in a pattern by the mould, usually of equal spacing. There is usually some water-equivalent material called bolus or build-up (BU) between the brachytherapy tubes and the skin; this enables the dose cloud, by the time it gets to the skin, to have a homogeneous wave front (see Figure 5). External beam radiotherapy or teletherapy is usually when there is a relatively large distance between the radioactive source and the skin; this is called the source skin distance (SSD). At this distance the radiotherapy is attenuating slowly; therefore, from one point to another there is only a small drop in the intensity of radiotherapy measured. EBRT is therefore ideal for irradiating a volume in a homogeneous way.

Brachytherapy has the advantage of being able to irradiate large areas. Disadvantages include the need for expert construction and skill within the Radiation Department. There needs to be great care with daily setup as there can be inaccuracies in putting the mould onto the patient and this can significantly affect the dose delivered. This is particularly so if the patient changes contour during treatment, for example, loses weight. One also has to be aware that there is a low dose of radiotherapy away from the brachytherapy source and so, when irradiating the scalp, organs such as eyes and hippocampus should be contoured on the planning scan so dose to these structures can be calculated. BT also has the disadvantage of needing a planning scan. BT is not always available in every department.

External beam radiotherapy is made up of either photons or electrons. Photons can be regarded as coming from a kilovoltage source, another name of this type of RT is superficial radiotherapy (SXRT), or from a mega volt source, usually from a linear accelerator, where this type of RT is known as mega volt therapy (MVT). MVT does require build-up (BU), a tissue equivalent material that ensures that the skin surface gets full dose.

SXRT has the advantage of ease of setup. Usually a mask is not needed. The radiation field can be further finessed by laying thin lead shields directly on to skin; this is called skin collimation. The patient can be visible with direct vision to the treating staff through lead glass, making it easier to treat patients that are cognitively impaired. The need for radiation protection is not as demanding as for MVT as the beams are of a much lower penetration. There are disadvantages to SXRT. It can give significant dose to a depth depending on the SSD. Usually there is a maximum field size of 8 cm. Not all departments have SXRT. We recommend the quality or strength of beam to use is 100 kVp which is usually equivalent to a half value layer (HVL) of 1 mm of aluminium. This gives enough penetration to the skin at depth to ensure that skin appendages are covered.

Grenz rays in our opinion should not be used as the penetration is not sufficient and lentigo maligna in skin appendages cannot be sterilised, leading to in-field recurrence [12, 13]. The maximum dose of the beam (Dmax) is dose on the skin, otherwise known as the incident dose (ID). The minimum dose at 5 mm into tissue should be 80% of the incident dose, and this value will be found on the depth dose curves which are specific to each machine. The shorter SSD of SXRT makes this technique ideal for treating concave surfaces such as the inner canthus. The inverse square law shows clearly that, at a short SSD, the concavity of the wave front is much more acute than at a distance. Therefore, the wave front of SXRT is ideal to treat concavities, for example, the inner canthus and nasal alar groove. This is compared to the wave front of mega volt therapy, with an SSD on one metre, when incident on skin. The wave has a much flatter profile and is better for irradiating larger fields of skin to a homogeneous dose.

**Figure 6:**
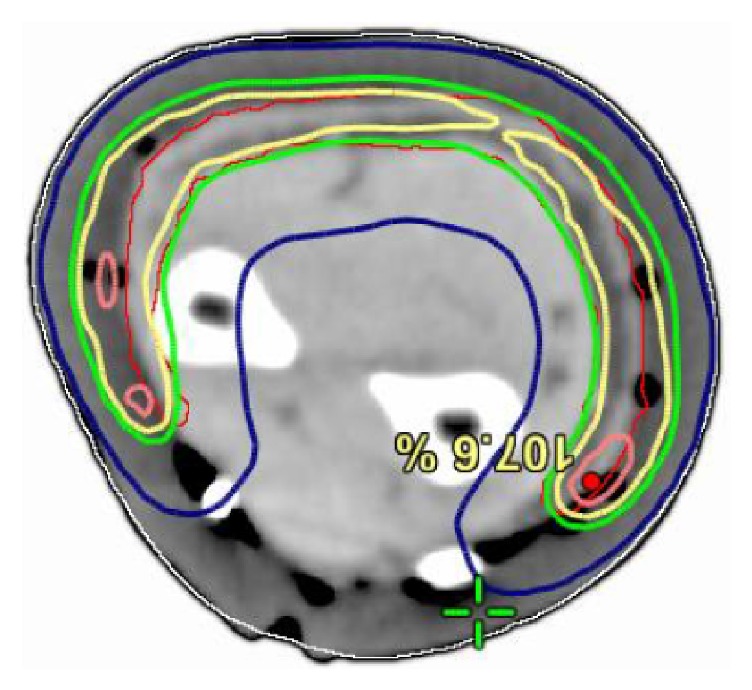
VMAT allows treating of a convex forearm, leaving a strip for lymph flow.

Besides SXRT and Grenz Rays, EBRT photons can be generated by linacs and are called MVT photons. The newest technique in MVT photons is volumetric modulated arc therapy (VMAT), which is the latest iteration of intensity modulated radiotherapy (IMRT). This is dosed as per ICRU 83. [14]. MVT with three-dimensional conformal radiotherapy (3D-CRT) will not be mentioned in this article. The advantage of VMAT is that it can deliver a homogeneous dose to a volume with rapid fall-off; that is, there is good conformality of dose to the volume needed to be treated. It is also quick to deliver [15] and is ideal therefore for field therapy, which could be defined as treating skin fields over 50 cm^2^ in size. The SSD is usually around a metre and therefore is excellent for giving uniformity of dose at the beam front over a large area. It can treat convexities, particularly those that are long or large, for example, on scalps, foreheads, arms, and legs (Figure 6). The disadvantages of VMAT are that it does require a planning CT, one needs to consider the dose to deeper structures, expertise is needed in planning, and physics quality assurance is needed. Meticulous care is needed in daily setup and attention to detail for patient immobilisation. VMAT does require bolus to ensure full dose to skin and there can be issues with air gaps and so attention to detail in treatment is also needed.

Another type of EBRT is electrons. These also come from a linear accelerator. Electrons are commonly dosed to the 90% isodose [16]. The advantage of electrons is that they can also treat a greater area of skin. Electrons from a modern linear accelerator can treat an area 20 x 20 cm in size. There are several disadvantages with electrons. They do require a planning scan and they also need BU to get full dose on skin; the thickness of BU will depend on the energy being used and the obliquity of the beam. Usually, the thickness needed is between 0.5 and 1 cm. The use of BU increases the setup time and the uncertainty of treatment.

**Figure 7:**
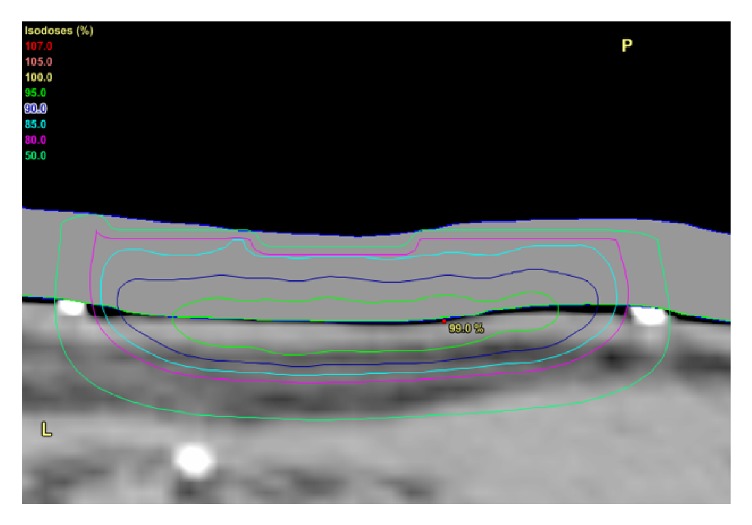
Electrons do best when a flat surface is treated with a beam perpendicular to the surface. Here in this image the 90% isodose line, which is the line that is the dose prescription line, straddles the skin surface, a good plan. The wires are marking the field. The dose drop-off towards the subcutis is ideal.

**Figure 8:**
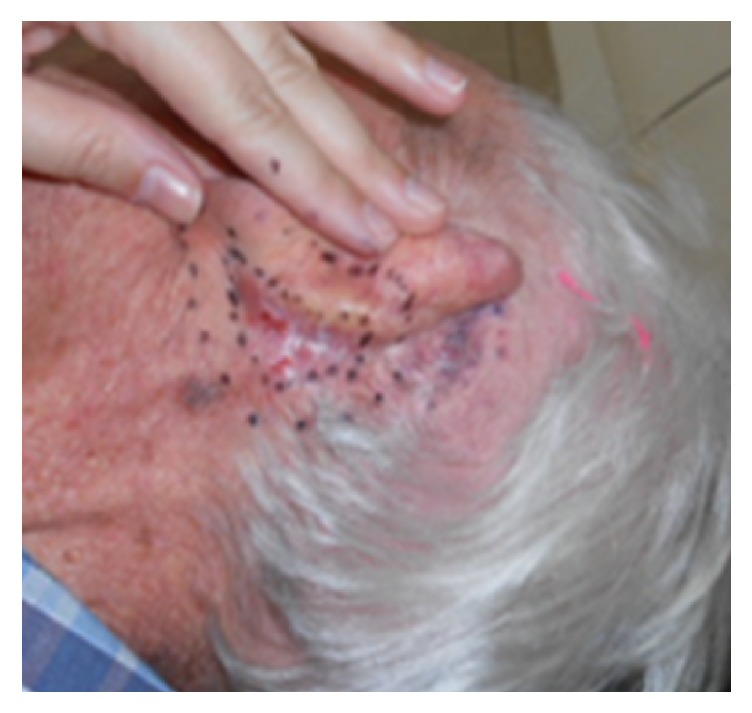
Electrons do prefer a flat field. Oftentimes the fields needed to be treated are modified in order to make them flat. For example, the ear can be taped forward in order to render flat the lesion that spreads from medial surface of the pinna to the lateral skin of the scalp.

Electrons do prefer a flat field that is perpendicular to the beam (see Figure 7). Oftentimes the fields needed to be treated are modified in order to make them flat. For example, noses can be taped flat to the side when treating the nasal ala. One can tape the ears forward in order to treat the medial surface of the pinna (see Figure 8). One can also use bolus material to make the field as flat as possible to the incident beam, for example, filling a conchal bowl with solid water-equivalent material. Electron dosimetry can be difficult when treating inhomogeneities, for example, the nose with air tissue interfaces in the nostril and in sinuses. The treatment staff need to pack the nostril so it appears as a solid organ to the beam. This increases time on bed and uncertainties in the treatment.

**Figure 9:**
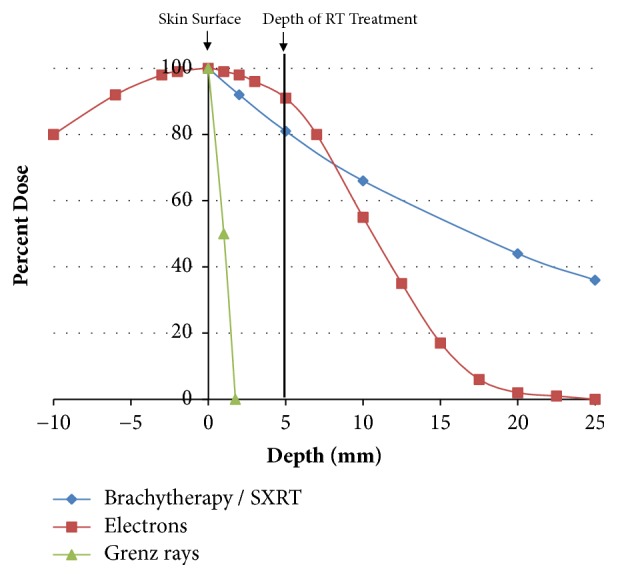
The depth of skin is on the X axis, the amount of radiotherapy is on the Y axis. Brachytherapy and superficial radiotherapy give good dose to skin surface and at 5 mm but do give significant dose to dermis which may lead to increased late effects, for example, fibrosis, telangiectasia in the future. Electrons do give a good drop-off in dose so there is less dose to dermis.

These different modalities have different depth dose characteristics which are displayed on the depth dose figure (see Figure 9). BT and SXRT give good dose to skin surface and at a depth of 5 mm but do give significant dose to dermis which may lead to increased late effects, for example, fibrosis and telangiectasia in the future. Electrons do give a good drop-off in dose so there is less dose to dermis but they do need BU on the surface. One must ensure there is no air gap between the BU and the skin, as electrons dosimetry is difficult to predict with inhomogeneities of density of material that it passes through.

### 5.3. Dose and Fractionation

The radiation oncologist then needs to describe a dose. Total dose, number of fractions, and therefore dose per fraction and length of treatment need to be determined. Patient factors will have an impact on these.

Fractionation ensures that late normal tissue fibrosis is minimised. However, longer fractionation requires multiple, even weeks of daily visits to the Radiotherapy Department, and can be the reason why patients decline radiation treatment. This is particularly true for the populations that have lentigo maligna who can be older, have challenged mobility, have comorbidities, and are dependent on others, for example, family and public providers for transport.

Increasing dose per fraction will increase normal cell death and replace it by fibrous tissue with the corresponding detriment in functional and cosmetic outcomes.

Dose fraction options for lentigo maligna are outlined in Table 1. This table is a table done based on biologically equivalent doses (BED) [17] (see Table 1).

The radiation oncologist then has to accept the best plan. Doses to critical structures including lens, orbit, optic nerve, lacrimal gland, parotid regions, etc. should be documented and noted to be below departmental tolerances for the plan to be accepted. Compromises should be detailed. Dose to CTV should fulfil criteria of ICRU 50, 83, and 37 [11, 14, 16].

## 6. Simulation

Simulation is part of the planning process. At simulation it has to be ensured that the patient consent has been signed and that the patient understands the rationale, process, and side effects of treatment. The necessary preparations for treatment should have been done by this stage, for example, seeing a dentist if any salivary gland is within the entrance or exit beams. The radiation oncologist should be armed with the knowledge of the CTV needed to be marked out. This will depend on having access to mapping from the dermatologist which could include RCM, dermoscopy, and targeted biopsies.

With the patient in the treatment position, the radiation oncologist marks on the CTV on skin with a water soluble texta and a template is constructed (see Figure 3(b)). Photos are taken of the CTV in the treatment position. An immobilisation device is made depending on the body part being irradiated. The texta marks are wired and perhaps have to even be put onto the immobilisation device like a mask so that they can be seen when the planning scan, usually a Computed Tomogram (CT), is done. More photos of the treatment that are set up with immobilisation devices in the treatment position are taken for verification at treatment setup. Close-up photographs of the CTV are required to enable recognition of where the area is on the patient with both the mask on and the mask off.

The CT protocol is then followed. A CT protocol with total CT scanning 10 cm above and 10 cm below the marked CTV is appropriate. More volume should be done if dose limiting organs are not completely within the field, for example, lungs. CT cuts at 2 mm spaces are appropriate.

The CT data set is then loaded into the planning computer and a treatment planning system is activated so the radiation oncologist can contour. The radiation oncologist should put a suitable margin on the CTV to create the PTV depending on department protocol and the modality being used. The PTV for VMAT is often 10 mm from the CTV for the body and 3 mm for the head and neck. The field edge is often 10 mm on CTV for electron treatments. The field edge is at least 5 mm on CTV for SXRT. The radiation oncologist should also ensure that organs at risk are contoured appropriately. With head and neck VMAT or brachytherapy, this includes salivary glands, lacrimal gland, and hippocampi as well as other dose limiting structures including lens, eyes, et cetera.

## 7. Treatment

When the plan is accepted, treatment can take place. This should preferably be delivered five days a week with no more than four days' break during treatments. A minimum of three fractions per week should be administered for lentigo maligna. On-treatment reviews are usually done weekly. If a patient has any of the gastrointestinal tract including mouth in the integral radiotherapy volume, it is important that they are weighed every week to ensure adequate nutrition; if the nutrition is compromised, patient contour can suffer and normal cells will not repair between the fractions, leading to an increase in acute effects. Nutrition enables normal cell repair between the fractions. OTRs should also be done by the radiation nurse to supervise skin reactions and care.

### 7.1. Skin Care during Radiotherapy

The most radiosensitive parts of the skin are the hair follicles and sweat glands. These provide natural moisture; therefore patients of radiotherapy to skin should use moisturizer from the beginning of their treatment within the treatment area as this may delay the onset of acute normal skin effects.

**Figure 10:**
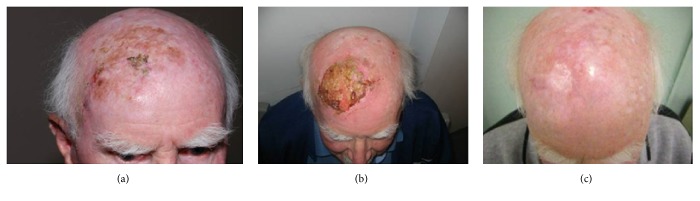
(a) This gentleman with a large 10 x 10 cm deposit of lentigo maligna on the right anterior scalp that had already grown into significant invasive melanoma. He was treated with external beam radiotherapy alone. (b) In the next figure, the acute reaction can be seen. He has an acute grade 3 reaction of wet desquamation. (c) Six months later, the final photo shows all the lentigo maligna has responded.

As radiotherapy progresses, the treatment area will develop erythema (grade 1), then dry desquamation (grade 2), and then wet desquamation (grade 3) [18] (see Figure 10). The side effects are from the inflammation caused by irradiation and even though swabbing will grow an organism, the inflammation is not necessarily cellulitis and oral or topical antibiotics may not help and may even be harmful.

**Figure 11:**
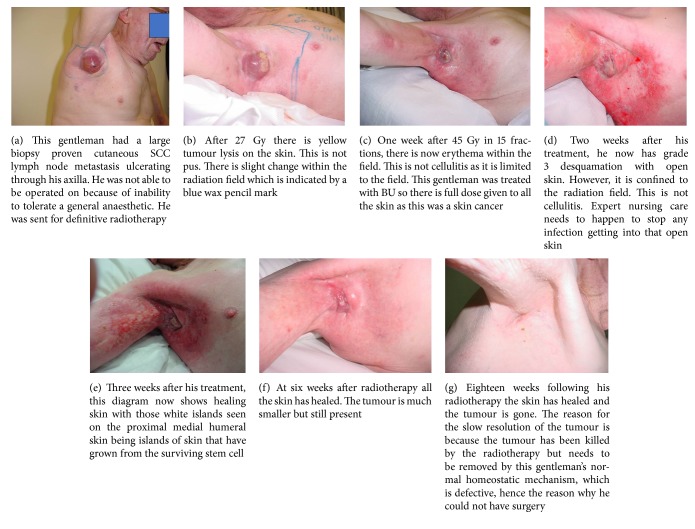
Case study of timeline of acute effects following radical radiotherapy to the skin.

With standard fractionation (2Gy per fraction) the skin usually closes two weeks after radiotherapy. Erythema can be noted by the patients for up to two months. The shorter the course of radiotherapy is, the more the side effects will lag behind the radiotherapy delivery. These side effects may not peak until after the radiotherapy has ceased (see Figure 11). At this stage moisturizer is usually replaced with a moist barrier cream that provides a matrix for new cells to grow over, for example, benzoyl peroxide. Expert nursing care helps prevent the open skin from becoming a portal to infection.

Late effects are avoided with adequate fractionation. Late effects represent the replacement of dead normal cells that have failed to repair radiation damage by fibrous tissue. The worst side effects are hypopigmentation, telangiectasia and cicatrization (see Figure 1), and hyperpigmentation and in particular loss of elasticity in the skin field treated. This can result in loss of nasal flaring, ectropion, et cetera.

Frequently, these late effects can be of cosmetic significance to the patient. One study of patients irradiated for cutaneous neoplasia involved 2,474 examinations of 1149 irradiated fields [19] and showed that after a minimum of 90 days after therapy, hypopigmentation was found in 64.7% of examinations, telangiectasias in 43.1%, and hyperpigmentation in 16.8%.

### 7.2. Other Side Effects during Radiotherapy

Care is taken to ensure that radiotherapy is only delivered to sites that need it. However, due to limitations of the treatment modalities and the closeness of radiation sensitive normal structures, bystander effects can happen.

Examples include unwanted therapy of the nasal mucosa in the exit beam when irradiating the tip of nose. In this case, nasal packing and use of intranasal shields help to decrease irritation but do increase set up time. One lotion used is a vitamin E in an oil base cream. It comes in a nasal shaped dispenser. This can help alleviate symptoms of acute radiation inflammation in the nasal mucosa when it starts to happen. Bleeding of the nasal mucosa is common and is helped also by Vaseline applied to the bleeding point on the patient's finger.

Irradiation of the oral mucosa can occur when irradiating the lip and an intraoral shield can be placed to alleviate this effect. Irradiating a cheek may cause xerostomia due to parotid irradiation but this can usually be avoided by a close attention to radiation dose being used and the dose volume histogram of the parotid gland.

Irradiating the nail matrix when irradiating the terminal phalanx can result in a lack of nail growth. There are various ways of decreasing the acute and late effects of radiotherapy in these situations with a general knowledge in the radiotherapy community.

## 8. Follow-Up

Follow-up should be done by the Multidisciplinary Team. The dermatologist should need to determine lentigo maligna clearance at six months. Radiation side effects can cause a false positive if this is attempted before that time

If a recurrence is suspected it should be biopsy proven. It needs to be assured that the recurrence is within the field or in the field edge or beyond the field. This could be done by the use of a template (see Figure 3(b)). The biopsy can give a clue to whether the recurrence is in-field. RT effects such as hyalinization and fibrosis should be seen in the stroma if there is in-field recurrence. In-field recurrence of lentigo maligna can be salvaged by surgery.

Further radiotherapy for in-field recurrence is usually not possible. It may be possible after many years of the previous radiotherapy, particularly if the previous radiotherapy was given to a dose that was considered low for lentigo maligna and was given in a fractionated manner and late effects are not clinically apparent in the proposed field of retreatment. Salvage can also be done by crossing over to Aldara or just by close observation, giving treatment when an invasive malignancy occurs. This is particularly so in older patients who may not be able to handle more acute toxicity.

## 9. Special Cases

There are very few cases that cannot be treated with radiotherapy. One case is radiotherapy to lentigo maligna of the conjunctivae on the globe. This could be treated with brachytherapy. There could also be an unsealed source solution to this. Both would be considered experimental.

### 9.1. Immunosuppressed Patients

Immunosuppressed patients can be defined as those having human immunodeficiency virus (HIV), a history of transplant, chronic lymphatic leukaemia, being on azathioprine, and having a chronic dose of steroids over 7 mg of prednisolone equivalent daily. Radiotherapy may cause some antitumour effect by immune stimulation. Immunosuppressed patients may therefore require more dose. On the other hand, there is anecdotal evidence to suggest that those who are immunosuppressed because of the immunosuppressing drugs may be rendered more radiosensitive by these drugs and may require less therapy. Radiation sensitizing drugs used in immunosuppression include azathioprine, methotrexate, and hydroxy urea. Caution should be used during the on-treatment review process to monitor how these patients are progressing in terms of normal skin reactions to radiotherapy and the treatment finessed as needed.

### 9.2. Poorly Vascularised Tissue

Normal cells require good oxygenation and nutrition to heal between fractions, so caution needs to be taken when dealing with poorly vascularised tissue, for example, in some patients in the lower legs. Fractionation may need to be even further drawn out, for example, using 1.8 Gy per fraction. Areas covering multiple surgeries in the past may be hypoxic and needing more dose. Care needs to be taken in radiation therapy field design as skin rotation may have spread lentigo field change to different zones and even below into the patient's dermis. Recurrent lesions after ablative topical therapy may also need dose intensification.

### 9.3. Lentigo Maligna after Surgery

**Figure 12:**
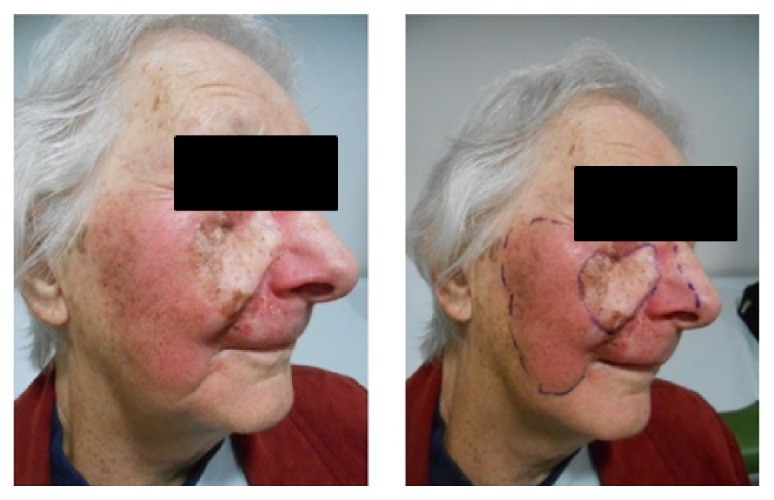
Recurrent LM postsurgery at a dose of 50 of 60 Gy to skin the flap from the lower anterior right neck on face does not get an acute effect. This lady had LM and a large flap to the right medial cheek had failed to clear it. Salvage RT was given. At 50 Gy the surrounding skin is red but the flap is not. The mechanism behind this could be that the flap is relatively hypoxic to the skin and therefore does not exhibit radiation side effects like the normal skin does.

LM postsurgery may be visible on RCM or dermoscopy. Previous surgery may cause hypoxia and RT dose may need to be intensified (see Figure 12). When adding radiotherapy to previous surgery, lymphoedema needs to be considered. Both modalities are unkind to lymphatics.

### 9.4. Lentigo Maligna of the Eyelid

**Figure 13:**
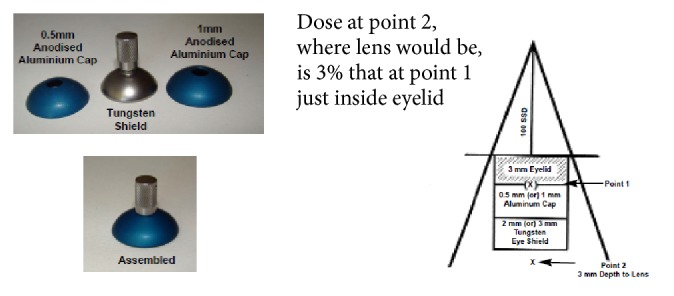
Internal eye shield design.

**Figure 14:**
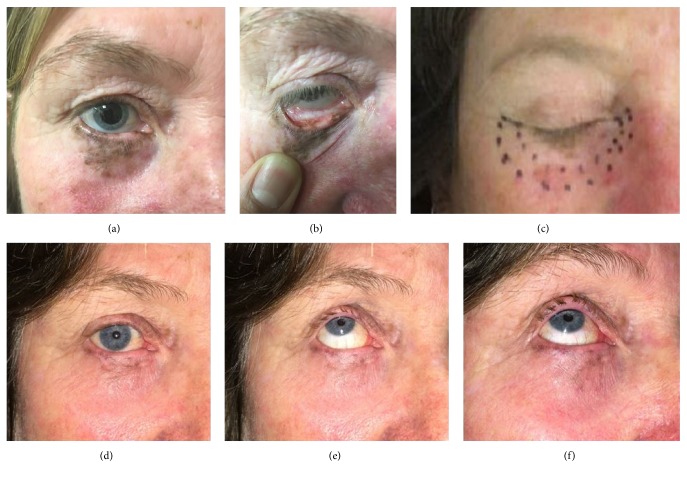
(a, b) This lady had a significant pigmented lentigo maligna of the lower right eyelid. It spilt over onto the palpebral surface of the conjunctiva. (c) The next figure shows the CTV as marked by the radiation oncologist and then a 5 mm expansion to superficial radiotherapy field. (d, e, f) The following three images are photos taken six weeks after treatment. One can see significant resolution of the lentigo maligna and loss of the lower lid eyelashes but the globe is untouched. Eye and vision are fine as at baseline.

Lentigo maligna of the eyelids and palpebral conjunctiva can be treated using internal eye shields (see Figures 13 and 14). This involves daily local anaesthetic drops in the eye and insertion of an internal eye shield. The internal eye shield is inserted between eye lid and globe to protect the globe. It is inserted under local anaesthetic. An eye patch should be worn for one hour after treatment so as to protect the eye until sensation returns. Prophylactic local chloramphenicol ointment can be used. Those with dry eyes can use a natural lubricant to assist in the eye shield deployment. An experienced radiation therapist will ensure that there is no trauma to the globe on insertion. This may be done by a nurse. Any corneal pain necessitates a treatment break and ophthalmological review. Eyelashes will unfortunately be lost during the radiotherapy. Hair will usually grow back within a year following high-dose radiotherapy.

### 9.5. Mucous Membrane of Mouth and Nose That Is in the Radiation Field

When there is lentigo maligna of the vermilion lip or nasal ala, it is still possible to use radiotherapy. Lead shields are used to decrease the dose to the mucosa. These shields are special. They are made of lead in wax, as the wax decreases the backscatter from radiotherapy incident on the lead. With the nose, the shield is placed on the medial septal wall and kept in place with BU material. This means that any backscatter will go into the BU rather than into the nasal ala if the shield was placed laterally. BU material can also be used to separate lips from teeth when irradiating the lip.

### 9.6. Ears and Electrons

BU material can be used to make the surface of the pinna flat when treating the pinna with radiotherapy. One can actually treat through the pinna to a posterior pinna lesion, using the pinna as BU. Water-equivalent material is put down the external auditory canal to absorb dose and avoid inhomogeneities resulting from the beam going through media interfaces of different electronic densities, for example, flesh to air to bone which can happen as the beam traverses the sinuses around that part of the anatomy.

## 10. Conclusion

LM can be treated in a definitive way with RT. This article tries to explain the RO experience gained at a high volume site involved with an international trial. The purpose of the review is to pass on that experience to other centers who may want to join the trial, especially to supplement the experience of local coinvestigator radiation oncologists. The review covers decisions that need to be made in RT in LM and also helps delineate the various ways of treating different parts of the anatomy that suffer from lentigo maligna with radiotherapy. The review follows in outline the different decisions required of ROs in the treatment process. It also covers how to manage side effects and other common scenarios including LM in immunosuppressed patients, in poorly vascularised tissue, postsurgery, of the eyelid, and of mucous membrane of mouth and nose that are in the radiation field.

## Figures and Tables

**Table 1:** Biologically equivalent dose (BED) doses for lentigo maligna.

Total dose (Gy)	No of fractions / weeks of RT	Dose per fraction	BED^*∗*^ early	BED^*∗∗*^ late	EQD2^*∗∗∗*^ *α*/*β* = 10(Gy)	EQD2^*∗∗∗*^ *α*/*β* = 3(Gy)
60	30/6	2	72	100	60	60
55	25/5	2.25	68.91	98.44	56.15	57.75
50	20/4	2.5	62.5	91.67	52.08	55
45	15/3	3	58.5	90	48.75	54
40	10/2	4	56	93.33	46.67	56

^*∗*^Biologically effective dose of early responding tissue using *α*/*β* of 10. ^*∗∗*^Biologically effective dose of late responding tissue using *α*/*β* of 3. ^*∗∗∗*^Equivalent dose in 2Gy fractions (EQD2). Biologically effective dose (BED) = n x d(1 + d/*α*/*β*). n = number of treatment fractions. d = dose per fraction in Gray (Gy). *α*/*β* = dose at which the linear and quadratic components of cell kill are equal.
